# Contract award criteria in public procurement procedures – The possibility of improving the situation of society from the perspective of the European Union and Poland

**DOI:** 10.12688/f1000research.153917.2

**Published:** 2024-09-10

**Authors:** Mateusz Brzeziński

**Affiliations:** 1 Master of Laws (LL.M. and magister), University of Warsaw, Warsaw, Masovian Voivodeship, Poland

**Keywords:** public procurement, tenders, contract award criteria, socially responsible public procurement, social aspects

## Abstract

**Background:**

Public tenders are vital for a country’s GDP and citizens’ quality of life, enabling public administration to achieve various goals. Developing and developed countries allocate over 10% of their GDP to public procurement. This highlights the significant societal support public tenders can provide, making it important to consider how they can further benefit society.

**Policy and implications:**

Public procurement can achieve policy objectives and benefit society by selecting tenders based on criteria beyond price, such as economic advantage and social benefits. This approach, endorsed by EU directives since 2014, encourages innovation and socially responsible practices. Contracting authorities in the EU can use social, environmental, and qualitative criteria to determine the most advantageous offers.

Poland’s Public Procurement Law (PPL) allows contract award criteria based on quality and price, including social aspects. Contracting authorities can specify criteria like employing marginalized groups, though these criteria often face scrutiny and legal challenges. Non-price criteria aim to enhance competition and achieve social, environmental, and economic goals.

**Recommendations:**

EU and Polish laws permit and encourage using social aspects as contract award criteria in public procurement. However, contracting authorities must analyze priorities, risk balancing, time constraints, and departmental coordination to effectively implement these criteria. This approach can improve the social situation and support specific groups.

**Conclusions:**

Public procurement significantly influences a country’s economy and quality of life, with EU and Polish laws allowing social criteria in contract awards. Directive 2014/24/EU supports tenders based on economic and social benefits. Poland’s PPL aligns with this, emphasizing marginalized group employment. Effective implementation fosters job creation, social integration, and improved living standards.

## Introduction

There should be no doubt that public tenders are one of the most important elements not only of generating the GDP of a given country, but also affect the quality of life of citizens. By means of public tenders, public administration bodies can meet various types of goals. OECD countries spend on average nearly 13% of annual GDP on public tenders ( OECD 2021). EU Member States spend on average around 14% of annual GDP on public tenders. Poland spends on average 10% of its annual GDP in public tenders. It means that countries can significantly support the functioning of society, using the tools that public tenders can provide. Therefore, it is worth considering how these public tenders can improve the situation of society.

## Policy outcomes and implications

### Legal basis in the EU

According to Jolien Grandia and Joanne Meehan public procurement can be leveraged to accomplish specific policy objectives, thereby benefiting society. This approach often involves selecting a tender design that awards contracts based on criteria beyond just the price, such as choosing the tender that offers the greatest economic advantage. Numerous studies have found that public procurement can stimulate innovative solutions by procuring functions rather than predefined goods or services ( Geroski 1990; Elder and Georghiou 2007; Aschhoff and Sofka 2009). Socially responsible public procurement is an approach in which contracting authorities use solutions that allow them to achieve additional social benefits when awarding public contracts. In practice, contracting authorities in European Union Member States have been using solutions to achieve additional social benefits for over 30 years, but it was only in 2004 that they were included in the EU directives on public procurement.

One of such elements of socially responsible public procurement may be the contract award criteria. The essence of the contract award criteria, as emphasized in recital 92 of the preamble to Directive 2014/24/EU, is to enable a comparative assessment of the level of performance offered in each of the tenders for the subject of the contract. Bearing in mind also the principle that public tenders should be awarded on the basis of objective criteria ensuring compliance with the principles of transparency, non-discrimination and equal treatment, with a view to ensuring an objective comparison of the relative value of tenders in order to determine, in conditions of effective competition, which of the tenders is the most economically advantageous (see recital 90 of the preamble to Directive 2014/24/EU), all principles that the contracting authority intends to apply to the evaluation of tenders (i.e. criteria, weights and method of evaluation based on the criteria) must be specified in the procurement documents in an unambiguous and understandable manner. The description of the evaluation criteria and principles of evaluation of offers should therefore allow contractors to obtain full knowledge of how and based on what information their offers will be assessed, so that they can include appropriate data in the content of their offers.

The EU legislator himself in article 67 para 2 of Directive 2014/24/EU indicates that the most advantageous offer may include the best value for money, which is estimated on the basis of criteria including qualitative, environmental or social aspects related to the subject of a given public procurement - depending on which decision is made. The contracting authority itself will decide on the criteria.

It is important that the EU legislator advised contracting authorities that one of the criteria for evaluating offers may be social or environmental aspects ( Andhov et al. 2020). It is important because in the previously applicable Directive 2004/18/EC, in article 53 para 1 regarding contract award criteria, there was no indication at all that social aspects could be one of the possible contract award criteria. This also means that the EU legislator has noticed that it is actually possible to include this element in the contract award criteria and that they can not only help obtain the most advantageous offer in general, but it is also possible, using social aspects in public procurement, to meet the social goals of countries or local government units. Indeed, policies aimed at promoting societal objectives like social sustainability or innovation can impact the criteria ( Kadefors et al. 2021).

Nevertheless, it is noted that the lowest price is chosen when the goal is to maximize savings, while the best value approach is used for more complex projects ( Ballesteros-Perez et al. 2017). However, each time it is the contracting authority who must decide which contract award criteria to choose for a given public tender. Importantly, the EU legislator does not indicate exemplary contract award criteria - it describes them rather in terms of categories in which contracting authorities can look for appropriate solutions ( Caranta and Sanchez-Graells 2021). Therefore, the freedom of choice rests with the contracting authority both in terms of selecting the category of contract award criterion or criteria in general, as well as in terms of determining these criteria in detail.

### Legal basis in Poland

Due to the fact that the EU legislator decided to use directives to regulate the issue of public procurement in the EU, in Poland the issue regarding contract award criteria, at the level of principles and objectives, is the same as in the provisions of Directive 2014/24/EU. However, precisely because the issue was regulated by directives, EU Member States were able to clarify and detail certain issues. This also happened in the case of Poland, where the PPL regulations stated that the most advantageous tender may be selected on the basis of quality criteria and price or cost, according to article 242 para 1 point 1 of the PPL.

In this respect, it is worth paying attention to article 242 para 2 point 2 of the PPL, where it is indicated that quality criteria may be, in particular, criteria relating to the social aspects, including the social and occupational integration of persons referred to in article 94 para 1 (persons with disabilities, the unemployed, jobseekers, who do not remain in employment or do not perform gainful employment, to-be self-reliant persons, persons deprived of liberty or released from prisons having difficulties in integration with the environment, persons with mental disorders, homeless persons, persons who have obtained refugee status or subsidiary protection in the Republic of Poland, persons under the age of 30 and after 50 years of age with job-seeker status, without employment, persons who are members of disadvantaged minorities, in particular members of national and ethnic minorities). In this regard, it is worth mentioning that the Polish legislator, under certain circumstances, mandates the employment of individuals under an employment contract for the execution of the contract's subject matter - the legal obligation in this regard is provided for by article 95 paragraph 1 of the PPL. As indicated in the case law of the National Appeals Chamber, “The application of Article 95 of the PPL cannot lead to an unjustified restriction of competition by preventing the submission of bids from contractors who perform services personally or through subcontractors operating as sole proprietorships. Such an action distorts the idea of competition. The application of Article 95 of the PPL must be justified by the conditions of the specific procurement procedure, and the implementation of this provision in a particular procedure cannot be done in a blanket manner by merely repeating the content of the regulation. The contracting authority's task is to analyze the subject matter of the contract and the activities involved in its execution, and only after such analysis should it consider the potential application of Article 95 of the PPL” ( case KIO 154/24). Nevertheless, such a construction of the regulations means that the contracting authority cannot establish a contract award criterion related to the employment of individuals under an employment contract. Since the legislator, in certain situations, grants the contracting authority the right to require that work be performed by individuals employed under an employment contract, this cannot be transferred to contract award criteria (in other words, employment under an employment contract is associated with the description of the contract's subject matter and the absolute requirements related to its execution, whereas contract award criteria should account for something “additional”).

It is worth noting that while meeting the social aspects taken into account by the contracting authority in the conditions for participation in a procurement procedure or the terms of the contract (e.g. in terms of employment under an employment contract) is obligatory for the economic operator applying for a given contract, including them in the contract award criteria influences the selection of the most advantageous offer (the economic operator who meets the requirements regarding social aspects receives additional points in the offer evaluation), but failure to meet them does not, in principle, eliminate his offer from participation in the procedure. This is important because when determining the criteria for evaluating offers, these criteria can be defined more freely than when determining the conditions for participation in the procedure - because, in principle, they do not prevent the contractor from submitting an offer.

Before applying contract award criteria related to social aspects, it is advisable for the contracting authority to research the situation on the labor market in terms of the validity of the requirements specified in the criterion. This will avoid a situation in which the contractor gains an advantage in points by declaring the employment of e.g. unemployed people, and at the stage of order execution the declaration submitted as part of the offer turns out to be impossible to implement due to the lack of unemployed people able to perform the order. Depending on the decision of the contracting authority, related to the assessment of the specificity of the specific subject of the contract, as part of the social criterion, the contracting authority may specify the requirement to employ one or more people from one or several marginalized groups indicated in article 94 para 1 of the PPL. In practice, contracting authorities have doubts about how the evaluation is carried out in the case of the contract award criterion regarding the employment of a certain number of marginalized people to perform the contract. It is assumed that it is based on the economic operator’s only declaration regarding the employment of the indicated number of such people to perform the contract. The fulfillment of this obligation takes place during the implementation of the public procurement contract, and thus requires the contracting authority to determine the method of verifying the implementation of the obligation in question in the contractual provisions. In such a case, the contracting authority should specify the method of documenting and controlling the employment of marginalized people and the sanctions that will apply to the economic operator who does not implement the employment declared in the contract award criterion.

Nevertheless, Polish contracting authorities are quite reluctant to use contract award criteria related to social aspects. One can even risk the thesis that Polish contracting authorities are not willing to establish interesting and innovative contract award criteria. In my opinion, this is due to the fact that they are afraid of the potential use of legal remedies - an appeal to the National Appeals Chamber by a potential economic operator, and they also have doubts as to how such contract award criteria would be verified in an audit in projects co-financed from EU funds. Most often, in such a situation, a violation of the principle of fair competition and equal treatment of contractors is alleged. Nevertheless, in one of its judgments, the National Appeals Chamber emphasized that the contract award criteria are not intended to guarantee an “equal position of economic operators”, but to make competition between these economic operators more realistic. If the position of economic operators were to be equal, non-price contract award criteria would be illusory and essentially pointless. However, it is important that non-price contract award criteria are objective and guarantee that economic operators can compete. The competitiveness of economic operators in terms of non-price contract award criteria should be related to the objectivity of these criteria, so that their meaning and assessment is unambiguous, which in turn allows for a clear assessment of a given economic operator. Selection of non-price contract award criteria is the right of the contracting authority, which may also make the purpose of the public procurement procedure more realistic using these criteria, as well as obtain the subject of the contract of the highest quality ( case KIO 610/22). This means that contract award criteria by their nature - by promoting specific features or functionalities - lead to an increase in the chances of obtaining a contract by some economic operators, while limiting these chances for other economic operators.

However, it should be noted that these contract award criteria may allow the fulfillment of the fundamental principles of public procurement, which are included in article 17 para 1 point of the PPL, i.e. that the contracting body shall award the contract in a manner ensuring the best results of the contract, including social, environmental and economic effects, insofar as any of these effects can be obtained in a given contract in relation to the expenditure incurred.

### Actionable recommendations

None of the provisions of EU law and PPL prevent the use of social aspects as contract award criteria in public procurement procedures. Moreover, the provisions of relevant legal acts even encourage promoting the social aspects of public tenders through contract award criteria ( Caranta 2022). However, in order to apply the appropriate contract award criteria in this area, each contracting authority must perform appropriate analysis, including the following:

1) a matter of mentality ( Plaček et al. 2022) - moving away from the priority of price and the contract award criteria.
2) the issue of balancing risks ( Zhang et al. 2022) - challenging the contract award criteria criteria by potential economic operators vs. customer preferences.
3) a matter of time - the urgent need to prepare documentation of the proceedings, limiting the possibility of reliably preparing the criteria.
4) the issue of synergy of work ( Ochrana 2022) - the need to coordinate the work of the substantive and administrative and legal departments.

This means that each contracting authority has a chance, through public procurement procedures and contract award criteria, to improve the social situation and enable greater activity of specific groups of people.

## Conclusion

Public procurement plays a vital role in shaping the economic and social landscape of a country, impacting GDP and the quality of life of citizens.

The EU legal framework allows public procurement to support societal goals through the inclusion of social criteria in contract awards, as outlined in Directive 2014/24/EU, which enables contracting authorities to assess tenders based on more than just price, incorporating aspects that offer the greatest economic advantage and additional social benefits.

In Poland, PPL align with EU directives, allowing the selection of the most advantageous tenders based on quality and price or cost. Notably, Polish regulations also emphasize the inclusion of social criteria, such as the employment and integration of marginalized groups. While Polish contracting authorities are hesitant to use these criteria due to concerns about legal challenges and verification in audits ( Bajoori et al. 2024), there is a growing recognition of their potential benefits. However, this does not change the fact that for nearly 10 years, Polish authorities have been trying to influence the social aspect of public procurement. The first example of this was enabling the requirement for individuals performing activities within the framework of public contract execution to be employed under an employment contract. This was a step in the right direction, as one of the issues in the Polish labor market was related to employing individuals under different types of contracts (most often other forms of civil law contracts), which did not provide the same level of protection to employees as employment contracts did.

Public procurement offers a powerful tool for achieving social goals. By carefully selecting and implementing contract award criteria, contracting authorities can foster significant social benefits, contributing to a more inclusive and equitable society. Legal frameworks provide a solid foundation for such practices, though their success depends on the effective and innovative application by contracting authorities.

It should be noted that the solutions included in the EU and Polish legislation allow for achieving various social aspects and benefits, but they focus primarily on:

1) increasing employment stability and social security of employees,
2) employment and professional and social integration of people from groups at risk of social exclusion,
3) facilitating access to public procurement for social economy entities, and especially social enterprises conducting social and professional integration of people from groups at risk of social exclusion,
4) facilitating access to the subject of procurement for all users, in particular for people with disabilities and other special needs,
5) using products or services with social labels confirming compliance with standards regarding, among others, decent work or fair trade.

In the long term, the use of social public procurement contributes to:

1) creating jobs,
2) social and professional integration of people from groups at risk of social exclusion,
3) dissemination of decent work and fair trade standards among economic operators and employees,
4) development of social economy entities,
5) socializing the provision of services important to citizens, e.g. care services or revitalization services,
6) improving the quality of life, in particular of people with disabilities, by increasing their access to public spaces, buildings and services (adapting orders - services, construction works, purchased goods to the requirements of people with special needs).

## Data Availability

No data are associated with this article.
