# Supplementary material for: Contract award criteria in public procurement procedures – The possibility of improving the situation of society from the perspective of the European Union and Poland
Source: F1000Res. 2024 Sep 10;13:832. Originally published 2024 Jul 24. [Version 2] doi: 10.12688/f1000research.153917.2 (PMC11425036; doi:10.12688/f1000research.153917.2)
Supplement: Peer review history

# Reviewer report: Reviewer response for version 2

**DOI:** 10.5256/f1000research.171273.r322478

**Published:** 2024-09-25

**Authors:** Roberto Caranta

**Affiliations:** 1 Turin University, Turin, Italy

The author has duly implemented the suggestions. Congratulations

Does the paper provide a comprehensive overview of the policy and the context of its implementation in a way which is accessible to a general reader?

Partly

Is the discussion on the implications clearly and accurately presented and does it cite the current literature?

Partly

Are the recommendations made clear, balanced, and justified on the basis of the presented arguments?

Yes

Reviewer Expertise:

Comparative administrative law, EU law, public procurement

I confirm that I have read this submission and believe that I have an appropriate level of expertise to confirm that it is of an acceptable scientific standard.

# Reviewer report: Reviewer response for version 1

**DOI:** 10.5256/f1000research.168873.r315637

**Published:** 2024-08-26

**Authors:** František Ochrana

**Affiliations:** 1 Department of Public and Social Policy, Charles University, Prague, Czech Republic

I read with interest the text "Contract award criteria in public procurement procedures - The possibility of improving the situation of society from the perspective of the European Union and Poland." Thank you for this opportunity.

 The reviewed policy brief is a suitable contribution for the discussion of the issue of contract award criteria in public procurement procedures. The study contains a number of inspiring ideas on the evaluation criteria of public procurement. The analysis of this problem is carried out on the case of Poland. I accompany the assessment of the reviewed study with an additional comment. The comment contains topics for discussion as well as possible suggestions for the author for further research.

 Poland is an interesting CEE country that has a common historical footprint with the neighboring countries of the former Soviet bloc, such as the Czech Republic and the Slovak Republic. All three countries became new members of the EU on the same date (2004). They have adopted the same EU legislation for public procurement. We would therefore expect approximately similar behavior of bureaucrats in public procurement. In reality, however, when analyzing the contract award criteria in these countries, we see significant differences.

 Unlike other CEE countries, Poland has a low share of public procurement evaluations according to the lowest price. According to TED data (2018; 2024), Poland was one of the leading CEE countries in 2016, with about 16% of public contracts awarded on the basis of the lowest price. This is significantly different compared to the Czech Republic (82% in 2016; 64.9% in 2024) and Slovakia (85% in 2016; 89.6% in 2024) of orders evaluated according to the lowest price. It is an "odd strategy" in the selection of evaluation criteria (Pernica et al., 2024)(Ref-3).

 In the context of the theory of institutional changes (Mahoney and Thelen, 2010)(Ref-10) and historical institutionalism (Schmidt, 2008;(Ref-8),Schmidt, 2010(Ref-7); Plaček et al., 2022)(Ref-4), the question is raised, what are the causes of this difference?

 It is undoubtedly more factors. One of the key ones is legal regulations. The author of the policy brief deals with these regulations in the section Policy outcomes and implications (see part Legal basis in the EU and part Legal basis in Poland). I will try to respond to the author's inspirational ideas with the following note. I believe that on the basis of historical institutionalism and retrograde analysis (Ochrana, 2022)(Ref-2), we can explain the differences between Poland, the Czech Republic and Slovakia.

 The author of the policy brief focuses on the role of legal regulations of public tenders. These are contained in the new Polish Public Procurement Act ( https://www.portalzp.pl/ustawy/ustawa-z-dnia-11-wrzesnia-2019-r.-prawo-zamowien-publicznych-tekst-jedn.-dz.u.-z-2023-r.-poz.-1605-17500.html). To this I note:

 Act of 11 September 2019 - The Public Procurement Act has been amended several times since its adoption. The latest consolidated version of the law was published on 14 August 2023. (See https://www.portalzp.pl/ustawy/ustawa-z-dnia-11-wrzesnia-2019-r.-prawo-zamowien-publicznych-tekst-jedn.-dz.u.-z-2023-r.-poz.-1605-17500.html). This is a significant institutional change in the legal regulations of public tenders. The importance of this change in the Public Procurement Act was characterized by the then Minister for Enterprise and Technology, Jadwiga Emilewicz, by saying that contracting authorities would not only focus on the price offered, but would also take into account the quality of goods and services and their importance for the environment.

 When analyzing the Public Procurement Act, it is necessary to bear in mind that this legal regulation also has an impact on the behavior of bureaucrats in public procurement. In this respect, too, the reviewed policy brief is inspirational for public policy. Public procurement bureaucrats carry out their activities in a certain social and institutional environment, while the framework and incentives for their behavior are set by the public tender regulations.( https://ted.europa.eu/en/index )

 The author makes an interesting point that Polish contracting authorities are rather reluctant to use award criteria related to social aspects and that they are reluctant to establish interesting and innovative award criteria. The author explains this behavior by fear of the possible use of legal remedies (and appeal to a higher authority).

 This conclusion is in line with similar attitudes of contracting authorities in the Czech Republic, which we examined in the study by(Schmidt et al., 2016)(Ref-9). Advertisers are behaving opportunistically. I therefore support the author's assertion that contracting authorities are concerned about the possible use of legal remedies. By opportunistic behaviour, contracting authorities try to avoid the risk of a public tender failure and to reduce the risk of a public procurement review as much as possible (see Schmidt et al., 2016). At the same time, the bureaucrat is pre-cautious of risk and uncertainty. This caution stems from the fear of failure of the public contract. From the author's analysis, we can conclude that the behavior of bureaucrats in public procurement is characterized by risk aversion (see Tversky and Kahneman, 1992)(Ref-5); Bajoori, et al., 2024)(Ref-9). The bearer of risk aversion is the bureaucrat responsible for the administration of public procurement and for the formal contract. Formal contracts cannot completely avoid opportunistic behaviors (Zhang et al., 2022)(Ref-6). This explains why, in an institutionally underdeveloped environment, contracting authorities prefer to evaluate according to the lowest price.

 The author of the policy brief also points to the significant impact of the choice of evaluation criteria on public procurement as a tool for achieving social goals. This part of the policy brief is also inspirational for public policy. It shows that contracting authorities can promote important social objectives and contribute to a more inclusive and fairer society by choosing their evaluation criteria. This is in line with the above-mentioned idea of the influence of legal regulations on the conduct of contracting authorities. In a suitably set legislative environment, public procurement can thus become an important tool for the effective achievement of public policy objectives. This is a problem that, according to the author of the policy brief, four fundamental assumptions and conditions must be met to implement. These are mainly:

 1) a change of mentality that will lead to a change in behavior when selecting evaluation criteria ("moving away from the priority of price and the contract award criteria".)

 2) the issue of balancing risks;

 3) efficient administrative process (preparation of quality documentation);

 4) synergy of actors preparing and implementing public procurement.

 The author of the policy brief sees in the implementation of these conditions a chance for each contracting authority to "through public procurement procedures and contract award criteria, to improve the social situation and enable greater activity of specific groups of people."

 We can agree with this conclusion.  In short, it is mainly changes in the institutional environment and changes in the mentality of contracting authorities that can induce the necessary changes in the behavior of public procurement actors. In this respect, the conclusions of the policy brief are inspiring for other countries that are faced with a similar question of how to effectively achieve public policy objectives through appropriately set evaluation criteria for public procurement.

 Conclusion:

 In conclusion to the reviewed text "Contract award criteria in public procurement procedures – The possibility of improving the situation of society from the perspective of the European Union and Poland", I would like to state that it is an inspiring and stimulating policy brief. I recommend indexing it.  If the author finds in my evaluation some suggestions for improving his study, then I warmly welcome such an initiative.

Does the paper provide a comprehensive overview of the policy and the context of its implementation in a way which is accessible to a general reader?

Yes

Is the discussion on the implications clearly and accurately presented and does it cite the current literature?

Yes

Are the recommendations made clear, balanced, and justified on the basis of the presented arguments?

Yes

Reviewer Expertise:

public administration, public procurement

I confirm that I have read this submission and believe that I have an appropriate level of expertise to confirm that it is of an acceptable scientific standard.

## References

[1] : Risk aversion in first-price security-bid auctions. *Operations Research Letters* .2024;55: 10.1016/j.orl.2024.107121 10.1016/j.orl.2024.107121

[2] : Historical-evolutionary and Retrograde Approach to the Study of Social Phenomena and Public Administration. *NISPAcee Journal of Public Administration and Policy* .2022;15(2) : 10.2478/nispa-2022-0011 1-24 10.2478/nispa-2022-0011

[3] : Small arms procurement and corruption in small NATO countries. *Journal of Public Procurement* .2024;24(3) : 10.1108/JOPP-04-2024-0045 348-370 10.1108/JOPP-04-2024-0045

[4] : Historical institutionalism: a tool for researching the nonprofit sector in times of pandemic. *Innovation: The European Journal of Social Science Research* .2022; 10.1080/13511610.2022.2052027 1-15 10.1080/13511610.2022.2052027

[5] : Advances in prospect theory: Cumulative representation of uncertainty. *Journal of Risk and Uncertainty* .1992;5(4) : 10.1007/BF00122574 297-323 10.1007/BF00122574

[6] : Can Psychological Contracts Decrease Opportunistic Behaviors?. *Front Psychol* .2022;13: 10.3389/fpsyg.2022.911389 911389 10.3389/fpsyg.2022.911389 35719522 PMC9205657

[7] : Taking ideas and discourse seriously: explaining change through discursive institutionalism as the fourth ‘new institutionalism’. *European Political Science Review* .2010;2(01) : 10.1017/S175577390999021X 10.1017/S175577390999021X

[8] : Discursive Institutionalism: The Explanatory Power of Ideas and Discourse. *Annual Review of Political Science* .2008;11(1) : 10.1146/annurev.polisci.11.060606.135342 303-326 10.1146/annurev.polisci.11.060606.135342

[9] : An Empirical Analysis of Post-Contractual Behaviour in Public Works Contracts: The Czech Case as a Secondary Comparison with the Slovak Republic. *https://www.researchgate.net/publication/310773774_An_Empirical_Analysis_of_Post-Contractual_Behaviour_in_Public_Works_Contracts_The_Czech_Case_as_a_Secondary_Comparison_with_the_Slovak_Republic/citation/download* .2016;

[10] : Explaining Institutional Change. *https://www.cambridge.org/in/universitypress/subjects/politics-international-relations/comparative-politics/explaining-institutional-change-ambiguity-agency-and-power?format* .2009;

[11] : Institutions, Institutional Change and Economic Performance.1990; 10.1017/CBO9780511808678 10.1017/CBO9780511808678

# Response

**Published:** 2024-08-30

**Authors:** Mateusz Brzeziński

**Affiliations:** Not Applicable, Poland

Dear Professor,

 I would like to express my sincere gratitude for the insightful analysis and review of the article I prepared. I will incorporate the publications you mentioned in your review into my bibliography, as they indeed respond to the questions and issues highlighted in my article in an interesting and relevant manner.

 I hope that in the future we will have the opportunity to collaborate in the field of public procurement, and perhaps we can create a joint publication. It is extremely interesting to compare our countries in this regard – the challenges we face and how we attempt (or rather, do not attempt) to address them.

 Yours respectfully,

 Mateusz Brzeziński

# Reviewer report: Reviewer response for version 1

**DOI:** 10.5256/f1000research.168873.r307651

**Published:** 2024-08-14

**Authors:** Roberto Caranta

**Affiliations:** 1 Turin University, Turin, Italy

The article briefly highlights the framework for using award criteria in SRPP under EU and Polish law. It is clearly written, but for the phrase "Interestingly, under contract award criteria it is not possible to reward employment based on an employment contract. This is due  …". 

 It would benefit from articulating what are the social benefits we are talking about. 

 The reference list might be beefed up.

Does the paper provide a comprehensive overview of the policy and the context of its implementation in a way which is accessible to a general reader?

Partly

Is the discussion on the implications clearly and accurately presented and does it cite the current literature?

Partly

Are the recommendations made clear, balanced, and justified on the basis of the presented arguments?

Yes

Reviewer Expertise:

Comparative administrative law, EU law, public procurement

I confirm that I have read this submission and believe that I have an appropriate level of expertise to confirm that it is of an acceptable scientific standard, however I have significant reservations, as outlined above.

# Response

**Published:** 2024-08-22

**Authors:** Mateusz Brzeziński

**Affiliations:** Not Applicable, Poland

Dear Professor,

 Thank you very much for reading the article, reviewing it and providing tips.

 I will try to provide an improved version as soon as possible.
